# A Brain-Protective Sterol from Soft Coral Inhibits Lipopolysaccharide-Induced Matrix Metalloproteinase-9-Mediated Astrocytic Migration

**DOI:** 10.3390/biomedicines12010226

**Published:** 2024-01-19

**Authors:** Tsong-Hai Lee, Jiun-Liang Chen, Chuan-Hsin Chang, Ming-Ming Tsai, Hui-Ching Tseng, Yu-Chia Chang, Velayuthaprabhu Shanmugam, Hsi-Lung Hsieh

**Affiliations:** 1 Stroke Center and Stroke Section, Department of Neurology, Chang Gung Memorial Hospital, College of Medicine, Chang Gung University, Taoyuan 333, Taiwan; thlee@cgmh.org.tw; 2 Division of Chinese Internal Medicine, Center for Traditional Chinese Medicine, Chang Gung Memorial Hospital, School of Traditional Chinese Medicine, College of Medicine, Chang Gung University, Taoyuan 333, Taiwan; a12015@cgmh.org.tw; 3 Division of Basic Medical Sciences, Department of Nursing, Research Center for Chinese Herbal Medicine, Graduate Institute of Health Industry Technology, Chang Gung University of Science and Technology, Taoyuan 333, Taiwan; chuanhsin@mail.cgust.edu.tw (C.-H.C.); mmtsai@mail.cgust.edu.tw (M.-M.T.); hctseng@mail.cgust.edu.tw (H.-C.T.); ycchang03@mail.cgust.edu.tw (Y.-C.C.); 4 Department of Biotechnology, Bharathiar University, Coimbatore 641046, India; velayuthaprabhu@buc.edu.in; 5 Department of Neurology, Chang Gung Memorial Hospital, Taoyuan 333, Taiwan; 6 Department of Chemical Engineering, Ming Chi University of Technology, R&D Center of Biochemical Engineering Technology, New Taipei City 301, Taiwan

**Keywords:** soft coral, lipopolysaccharide, matrix metalloproteinase-9, cell migration, brain astrocytes, anti-inflammation

## Abstract

Matrix metalloproteinases (MMPs), which are proteolytic enzymes, promote blood–brain barrier (BBB) disruption, leading to neuronal damage and neuroinflammation. Among them, MMP-9 upregulation serves as an inflammatory biomarker in the central nervous system (CNS). Currently, the development of marine organism-derived bioactive compounds or metabolites as anti-inflammatory drugs has received considerable attention. The 9,11-secosteroid, 3β,11-dihydroxy-9,11-secogorgost-5-en-9-one (4p3f), is a novel sterol compound extracted from the soft coral *Sinularia leptoclado* with potential anti-inflammatory activity. However, the effect of and potential for brain protection of 4p3f on brain astrocytes remain unclear. Herein, we used rat brain astrocytes (RBAs) to investigate the effects and signaling mechanisms of 4p3f on lipopolysaccharide (LPS)-induced MMP-9 expression via zymographic, quantitative reverse transcription–polymerase chain reaction (qRT-PCR), Western blot, immunofluorescence staining, promoter–reporter, and cell migration analyses. We first found that 4p3f blocked LPS-induced MMP-9 expression in RBAs. Next, we demonstrated that LPS induced MMP-9 expression via the activation of ERK1/2, p38 MAPK, and JNK1/2, which is linked to the STAT3-mediated NF-κB signaling pathway. Finally, 4p3f effectively inhibited LPS-induced upregulation of MMP-9-triggered RBA cell migration. These data suggest that a novel sterol from soft coral, 4p3f, may have anti-inflammatory and brain-protective effects by attenuating these signaling pathways of MMP-9-mediated events in brain astrocytes. Accordingly, the soft coral-derived sterol 4p3f may emerge as a potential candidate for drug development or as a natural compound with neuroprotective properties.

## 1. Introduction

Astrocytes are one type of glial cells and the most abundant non-neuronal cell type in the central nervous system (CNS), comprising 19–40% of glial cells in the brain [1]. They exert a wide range of functions in the brain, including homeostatic maintenance and post-injury repair of the blood–brain barrier (BBB) in the CNS [2,3,4,5], as well as regulation of synapse formation and pruning, lactate levels, glutamate levels, and neuroinflammation in the brain [6]. However, the chronic inflammatory response of astrocytes triggers the migration of peripheral immune cells to the site of inflammation in the CNS, which further aggravates the inflammatory response and causes brain injury [7]. Astrocytes undergo reactive changes in morphology and molecular profile known as astrogliosis, leading to the release of proinflammatory molecules (including extracellular matrix, cytokines, and growth factors), which exacerbates the severity of brain injury [8].

Matrix metalloproteinases (MMPs), which comprise a family of calcium-dependent zinc-containing endopeptidases, play a role in the pathological processes of neuroinflammatory and neurodegenerative diseases [9]. MMP-9 serves as a potential disease biomarker in response to various physiological stimuli and pathological insults; the upregulation of MMP-9 enzymatic activity and expression contributes to a wide range of brain disorders, including BBB breakdown, epilepsy, schizophrenia, autism spectrum disorder, brain injury, stroke, neurodegeneration, pain, and brain tumors, among others [10,11].

Lipopolysaccharide (LPS) is a constituent found in the outer membrane of Gram-negative bacteria, and it is widely used as a potent proinflammatory agent in neuroinflammation studies [12]. Previous research has demonstrated that LPS induces the expression of MMP-9 in brain astrocytes and causes astrocyte migration via various signaling pathways, including phosphatidylinositol 3′-kinase (PI3K)/Akt, extracellular signal-regulated kinases (ERKs), and c-Jun N-terminal protein kinases (JNKs) [13], as well as nuclear factor kappa B (NF-κB) activation [14]. In addition to LPS, we previously reported that bradykinin (BK), a common proinflammatory mediator, upregulates MMP-9 protein expression through signal transducer and activator of transcription 3 (STAT3) activation in brain astrocytes [15].

Marine natural products (MNPs) are potent and promising sources of biologically active agents, which are extracted from marine organisms, such as tunicates, soft corals, and sponges [16]. Recently, research topics on MNPs and their potential in medicine have gradually attracted attention. The 9,11-secosteroid, 3β,11-dihydroxy-9,11-secogorgost-5-en-9-one (4p3f), which is isolated from the soft coral *Sinularia leptoclados*, can be structurally characterized by the C-9/11 oxidative cleavage of the C-ring [17,18,19]. Several studies have demonstrated that steroids from the genus *Sinularia* exhibit cytotoxicity and anti-proliferative effects against various cancer cells, including lung cancer cells, cervical adenocarcinoma, pancreatic epithelioid carcinoma, and promyelocytic leukemia cells [20,21,22]. Two novel 9,11-secosteroids, named sinleptosterols A and B, from the Taiwan soft coral *Sinularia leptoclados* have been reported to be compounds that have anti-inflammatory effects by inhibiting superoxide anion generation and elastase release in active human neutrophils [18]. Therefore, we believe that marine compounds have the potential to be developed as therapeutic drugs for inflammation-related pathologies. Herein, we investigated whether 9,11-secosteroid (i.e., 4p3f) exerts anti-inflammatory action in the CNS. In this study, the effect of 4p3f on LPS-induced MMP-9 expression in rat brain astrocytes and the underlying mechanism of action were examined.

In the current study, we demonstrated that 4p3f significantly inhibited LPS-induced MMP-9 expression in rat brain astrocytes (RBAs). Moreover, LPS-induced MMP-9 expression was mediated through phosphorylation of MAPKs (including ERK1/2, P38 MAPK, and JNK1/2), leading to the activation of STAT3-dependent NF-κB, which could be inhibited by pretreatment with 4p3f. Furthermore, pretreatment with 4p3f attenuated LPS-induced MMP-9-mediated RBA cell migration. These results suggest that the anti-inflammatory action of 4p3f (9,11-secosteroid) might be a potential therapeutic agent for brain inflammatory disorders.

## 2. Materials and Methods

### 2.1. Materials

Dulbecco’s modified Eagle’s medium (DMEM)/F-12 medium, fetal bovine serum (FBS), and TRIzol were purchased from Invitrogen (Carlsbad, CA, USA). Polyvinylidene fluoride (PVDF) membranes, enhanced chemiluminescence (ECL), and Western blot detection systems were obtained from GE Healthcare Biosciences (Chalfont St Giles, Buckinghamshire, UK). Anti-phospho-ERK1/2 (Cat#4377), anti-phospho-p38 MAPK (Cat#9211), anti-phospho-p65 NF-κB (Cat#3033), and anti-glyceraldehyde-3-phosphate dehydrogenase (GAPDH) (Cat#2118) antibodies were purchased from Cell Signaling Technology (Danvers, MA, USA). Anti-phospho-JNK1/2 and anti-STAT3 (Cat#sc-482) antibodies were purchased from Santa Cruz Biotechnology (Santa Cruz, CA, USA). Anti-mouse (Cat#7056) and anti-rabbit (Cat#5054) horseradish peroxidase-conjugated secondary antibodies were purchased from Santa Cruz Biotechnology (Santa Cruz, CA, USA). The iScript cDNA Synthesis Kit, SsoFast EvaGreen Supermix, and CFX Connect Real-Time Polymerase Chain Reaction (PCR) detection system were obtained from Bio-Rad Laboratories, Inc. (Hercules, CA, USA). A dual-luciferase reporter system was obtained from Promega (Madison, WI, USA). The inhibitors, including U0126, SB202190, SP600126, and Bay 11-7082, were purchased from Santa Cruz Biotechnology, and STAT3 inhibitor V (STAT3I) was obtained from Sigma-Aldrich Chemical, Co. (St Louis, MO, USA). All other enzymes, reagents, chemicals, and lipopolysaccharide (LPS) were obtained from Sigma-Aldrich (St. Louis, MO, USA).

### 2.2. Coral Material

The soft coral *Sinularia leptoclados* samples were collected from the coast of Pingtung, Taiwan. The samples were stored in a freezer at −20 °C until extraction. A specimen voucher was deposited at the Research Center for Chinese Herbal Medicine, Chang Gung University of Science and Technology, Taiwan (specimen No.: CGUST-C004-20.18-NOV).

### 2.3. Extraction and Isolation

The frozen samples were minced and extracted via maceration in ethyl acetate (EA) until exhaustion. The EA extract was subjected to repeated column chromatography on silica gel, and preparative RP-HPLC was used to obtain compounds of a steroidal nature. The structure of 9,11-secosteroids was deduced from a general spectroscopic analysis, including an examination of NMR spectra, and via comparison with data of related compounds [18].

### 2.4. Cell Culture and Treatment

The rat brain astrocytic cell line (RBA, CTX TNA2) was purchased from BCRC (Hsinchu, Taiwan). The cells were cultured in a DMEM/F12 medium containing 5% FBS, 100 IU/mL penicillin G, 100 mg/mL streptomycin sulfate, and nonessential amino acids at 37 °C under a humidified atmosphere with 5% CO_2_ according to a previously described standard protocol [15]. The cells were either untreated or treated with 4p3f or inhibitors of ERK1/2 (U0126), p38 MAPK (SB202190), JNK1/2 (SP600125), NF-κB (Bay11-7082), or STAT3 (STAT3I). These substances were added to the cell culture 1 h before the treatment of LPS (10 μg/mL) in all experiments. Treatment of RBAs with 4p3f, these inhibitors alone, or LPS stimulation had no significant effect on cell viability, as determined by an XTT assay. The cell passage numbers we used were from P10 to P15.

### 2.5. MMP Gelatin Zymography

Growth-arrested cells were incubated with different treatments for the indicated time intervals [15]. The cells were either untreated or treated with 4p3f or inhibitors of ERK1/2 (U0126), p38 MAPK (SB202190), JNK1/2 (SP600125), NF-κB (Bay11-7082), or STAT3 (STAT3I). After the treatments, the conditioned media were collected and analyzed by means of gelatin zymography, as previously described [15]. In briefly, the collected conditioned media were mixed with a 5× nonreducing sample buffer and analyzed on a 10% gel containing 0.15% gelatin. After electrophoresis, the gel was washed, incubated, stained, and then destained. The gelatinolytic activity appeared as parallel white bands on a blue background.

### 2.6. Total RNA Extraction and Real-Time RT-PCR Analysis

Total RNA was extracted from RBAs using the TRIzol reagent (Thermo Fisher Scientific, Waltham, MA, USA), and the concentration of total RNA was measured using a Nano100 Micro-Spectrophotometer (CLUBIO; Taipei, Taiwan) [15]. Then, cDNA was synthesized using an iScript cDNA Synthesis Kit and amplified on a spectrofluorometric thermal cycler (iCycler; Bio-Rad Laboratories). Real-time PCR was performed with the TaqMan gene expression assay system, using the primers and probe mixes for *COX-2* and endogenous *GAPDH* control genes. PCRs were performed using SYBR Green PCR reagents (Applied Biosystems, Branchburg, NJ, USA). The relative gene expression was determined based on the ΔΔCt method, where Ct denotes the threshold cycle. All experiments were performed in triplicate (*n* = 3).

### 2.7. Western Blot Analysis

Growth-arrested cells were incubated with different treatments for the indicated time intervals. After the treatments, the cells were washed with ice-cold phosphate-buffered saline (PBS), scraped, lysed in a lysis buffer, and centrifuged at 45,000× *g* for 1 h at 4 °C to yield a whole-cell extract, as previously described [15]. The samples were analyzed by means of Western blot, transferred to nitrocellulose membranes, and then incubated overnight with anti-phospho-ERK1/2, phospho-p38, phospho-JNK1/2, phospho-p65 NF-κB, phospho-STAT3, or GAPDH antibodies. The membranes were washed four times with TTBS for 5 min each and incubated with an anti-rabbit horseradish peroxidase antibody (1:2000) for 1 h. The immunoreactive bands were detected using ECL reagents and captured using a UVP BioSpectrum 500 Imaging System (Upland, CA, USA). The images were quantified and analyzed using the UN-SCAN-IT gel 6.1 software (Orem, UT, USA).

### 2.8. Immunofluorescence Stain

Cells were either untreated or pretreated with the inhibitors or 4p3f and then stimulated with LPS for 30 min, washed twice with ice-cold PBS, fixed with 4% (*w*/*v*) paraformaldehyde in PBS for 30 min, and then permeabilized with 0.3% Triton X-100 in PBS for 15 min. The cells were stained by incubating with 10% normal goat serum in PBS for 30 min, followed by incubating with an anti-NF-κB (p65) antibody (1:200 dilution (Cat#sc-372)) in PBS with 1% BSA at 4 °C overnight. On the following day, the cells were washed three times with PBS and incubated with a FITC-conjugated goat anti-rabbit antibody (1:150 dilution) in PBS with 1% BSA for 1 h. After three additional washes with PBS, the cells were mounted using an aqueous mounting medium. Images were acquired under a fluorescence microscope (Axiovert 200 M, ZEISS, Göttingen, Germany).

### 2.9. Promoter-Luciferase Reporter Gene Assay

The κB binding sites were cloned to the pGL4.32 (luc2P/NF-κB-RE/Hygro) vector containing a luciferase reporter system. All plasmids were prepared by using QIAGEN plasmid DNA preparation kits. These constructs were transfected into RBA cells by using a Lipofectamine reagent according to the instructions of the manufacturer. The transfection efficiency (~60%) was determined based on transfection with enhanced GFP. After incubation with LPS, the cells were collected and disrupted via sonication in a lysis buffer (25 mM Tris, pH 7.8, 2 mM EDTA, 1% Triton X-100, and 10% glycerol). After centrifugation, aliquots of the supernatants were tested for promoter activity using a Dual-Luciferase^®^ Reporter Assay System (Promega, Madison, WI, USA). Firefly luciferase activity was standardized for Renilla luciferase activity.

### 2.10. Cell Migration Assay

RBA cells were cultured to confluence in 6-well culture plates and starved with a serum-free DMEM/F-12 medium for 24 h. The monolayer cells were manually scratched using a pipette blue tip to create extended and definite scratches in the center of the dishes with a bright and clear field (~2 mm). The detached cells were removed, and a serum-free DMEM/F-12 medium was added with or without LPS after pretreatment with the inhibitors for 1 h, which contained a DNA synthesis inhibitor, hydroxyurea (10 μM), during the experimental period [15]. Cell migration was observed under a microscope and quantified by counting the number of cells from the resulting four-phase images for each point, as well as averaged for each experimental condition. The presented data are summarized from three independent assays.

### 2.11. Statistical Analysis

All data were analyzed using the GraphPad Prism Program (GraphPad, San Diego, CA, USA). Quantitative data were analyzed by means of one-way ANOVA, followed by Tukey’s honestly significant difference tests between individual groups. Data were expressed as mean ± SEM. A value of *p* < 0.05 was considered significant.

## 3. Results

### 3.1. The Effect of Soft Coral-Derived Sterol Extract on LPS-Induced MMP-9 Expression in Brain Astrocytes

**Figure 1:**
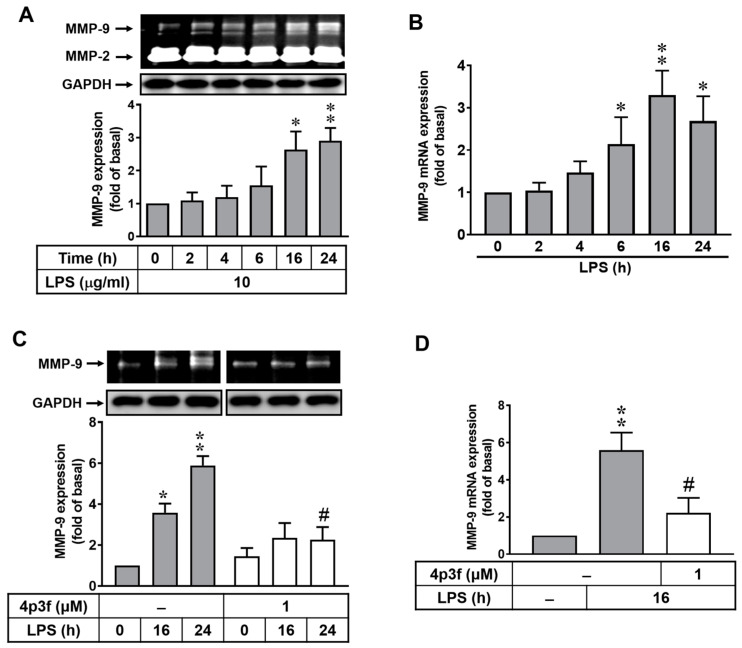
The effect of a soft coral-derived sterol extract on LPS-induced MMP-9 expression in brain astrocytes. (**A**,**B**) Cells were treated with LPS (10 μg/mL) for the indicated time intervals. (**A**) For MMP-9 expression, the conditioned media were collected and analyzed via gelatin zymography. And the glyceraldehyde-3-phosphate dehydrogenase (GAPDH) level of cell lysates (as an internal control) was assayed by means of Western blot analysis. (**B**) Total RNA was prepared and then *MMP-9* mRNA expression was analyzed via real-time RT-PCR. (**C**,**D**) Cells were pretreated with 4p3f (1 μM) for 1 h and then incubated with LPS for the indicated times. (**C**) The conditioned media were collected and analyzed for MMP-9 expression via gelatin zymography. (**D**) *MMP-9* mRNA expression was analyzed by means of real-time RT-PCR. Data are expressed as mean ± SEM (n = 3). * *p* < 0.05 and ** *p* < 0.01, as compared with the untreated control; ^#^ *p* < 0.05, as compared with LPS-treated cells only. The images represent one of three individual experiments.

An upregulation of MMP-9 is associated with a disruption of the blood–brain barrier (BBB) and neuroinflammation, which is a risk factor for brain inflammatory disorders, neurodegenerative diseases, cardiovascular diseases, and diabetes [23,24,25,26]. In this study, we first investigated whether LPS can induce MMP-9 expression in rat brain astrocytes (RBAs). Cells were treated with 10 μg/mL LPS for the indicated time intervals (0~24 h), and MMP-9 protein and mRNA levels were measured via zymographic and real time-PCR analyses. The results showed that LPS induced MMP-9 expression in a time-dependent manner, but not MMP-2 expression, with a significantly increased MMP-9 expression at 16 and 24 h (Figure 1A). Moreover, qRT-PCR was used to determine the effect of LPS on MMP-9 mRNA regulation. As shown in Figure 1B, LPS induced a significant increase in *MMP-9* mRNA expression after 6~24 h of incubation. The data revealed that LPS could induce MMP-9 expression in RBAs by upregulating the transcriptional and translational levels.

Previously, a study indicated that sterol extracts from soft coral have an anti-inflammatory ability [18]. Therefore, we further evaluated the effect of a soft coral-derived sterol extract of 4p3f on LPS-induced MMP-9 expression in RBAs. These cells were pretreated with 4p3f (1 μM) for 1 h, and then incubated with 10 μg/mL LPS for the indicated times (0, 16, and 24 h). The data showed that pretreatment with 4p3f significantly repressed LPS-induced MMP-9 expression in RBAs (Figure 1C). Moreover, LPS-induced MMP-9 mRNA expression was also inhibited after pretreatment with 4p3f in these cells (Figure 1D). These results suggested that the coral sterol extract of 4p3f may have a preventive effect against brain inflammation by attenuating LPS-induced MMP-9 expression in brain astrocytes.

### 3.2. 4p3f Attenuates LPS-Induced MMP-9 Expression by Blocking the MAPK Signaling Pathways

**Figure 2:**
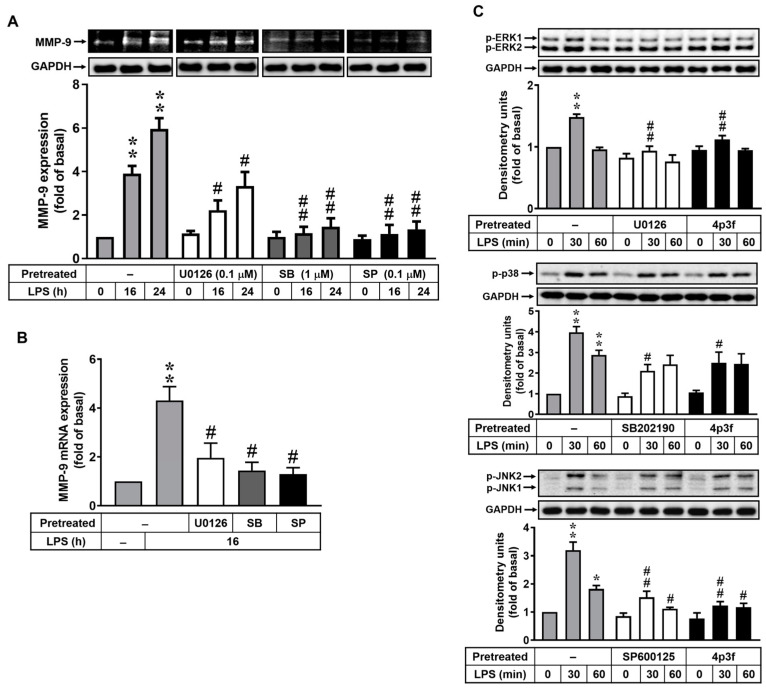
4p3f attenuates LPS-induced MMP-9 expression by blocking MAPK signaling pathways. Cells were pretreated with U0126 (0.1 μM), SB202190 (1 μM), or SP600125 (0.1 μM) for 1 h and then incubated with LPS (10 μg/mL) for the indicated time intervals. (**A**) For MMP-9 expression, the conditioned media were collected and analyzed via gelatin zymography. The GAPDH level of cell lysates was assayed by means of Western blot. (**B**) For *MMP-9* mRNA expression, total RNA was prepared and analyzed via real-time PCR. (**C**) Cells were pretreated with U0126 (0.1 μM), SB202190 (1 μM), SP600125 (0.1 μM), or 4p3f (1 μM) for 1 h and then incubated with LPS (10 μg/mL) for the indicated times. After treatment, cell lysates were collected and analyzed via Western blot for phosphorylation of ERK1/2 (p-ERK1/2), p38 MAPK (p-p38), JNK1/2 (p-JNK1/2), and GAPDH (as an internal control). Data are expressed as mean ± SEM (n = 3). * *p* < 0.05 and ** *p* < 0.01, as compared with the untreated control; ^#^ *p* < 0.05 and ^##^ *p* < 0.01, as compared with LPS-treated cells only. The images represent one of three individual experiments.

Activation of MAPKs regulates various functions of brain cells [27]. In addition, many reports have indicated that LPS induces MMP-9 expression via MAPK-dependent pathways in several cell types, including brain astrocytes [28,29]. Thus, to determine whether MAPKs also participated in LPS-induced MMP-9 expression in RBAs, cells were pretreated with inhibitors of MAPKs, including ERK1/2 inhibitor U0126 (0.1 μM), p38 MAPK inhibitor SB202190 (1 μM), and JNK1/2 inhibitor SP600125 (0.1 μM), for 1 h and then incubated with LPS (10 μg/mL) for 0, 16, or 24 h. As shown in Figure 2A, pretreatment with U0126, SB202190, or SP600125 significantly reduced LPS-induced MMP-9 protein expression in the cells. Moreover, LPS-induced *MMP-9* mRNA expression was also inhibited by pretreatment with U0126, SB202190, or SP600125 (Figure 2B). These data demonstrate that ERK1/2, p38 MAPK, and JNK1/2 signals are involved in LPS-induced MMP-9 expression in RBAs. To further investigate the effect of 4p3f on MAPK signaling pathways, a Western blot analysis was performed to examine the phosphorylation of ERK1/2, p38 MAPK, and JNK1/2 in cells exposed to LPS for 0, 30, or 60 min. The results showed that LPS could stimulate phosphorylation of ERK1/2 (upper panel), p38 MAPK (middle panel), and JNK1/2 (lower panel) in a time-dependent manner in RBAs, with a significant increase at 30 min (Figure 2C). Pretreatment with U0126, SB202190, and SP202190 significantly inhibited LPS-stimulated phosphorylation of ERK1/2, p38 MAPK, and JNK1/2, respectively. Moreover, LPS-stimulated phosphorylation of MAPKs was attenuated by pretreatment with 4p3f (Figure 2C). These results indicated that 4p3f could inhibit LPS-induced MMP-9 expression by attenuating activation of MAPK (i.e., ERK1/2, p38, and JNK1/2) signaling pathways.

### 3.3. 4p3f Inhibits LPS-Induced MMP-9 Expression by Reducing NF-κB Activation

**Figure 3:**
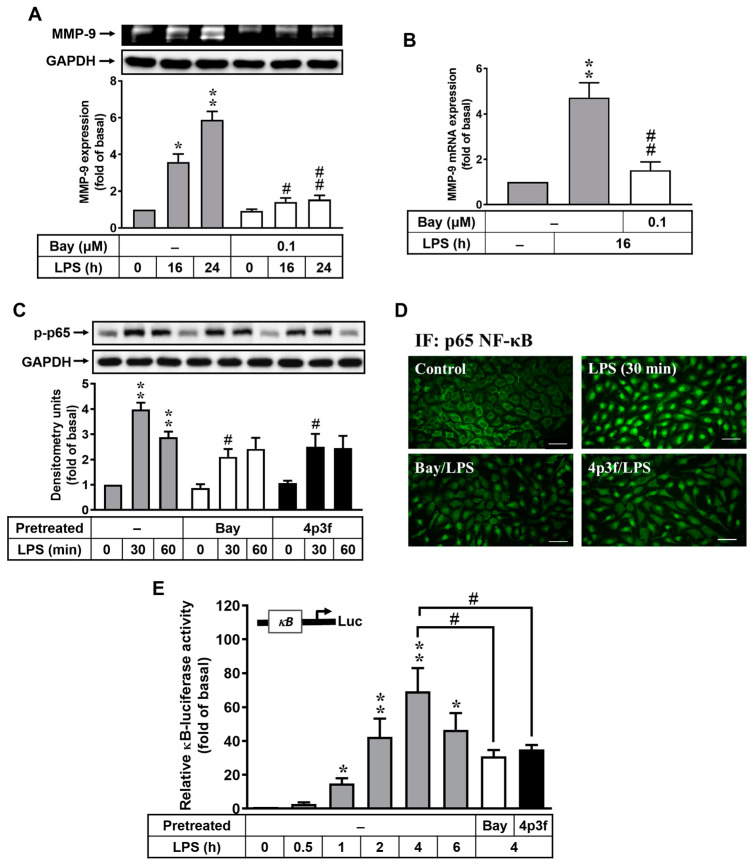
4p3f inhibits LPS-induced MMP-9 expression by reducing NF-κB activation. Cells were pretreated with Bay11-7082 (Bay, 0.1 μM) for 1 h and then incubated with LPS (10 μg/mL) for the indicated time intervals. (**A**) For MMP-9 expression, the conditioned media were collected and analyzed via gelatin zymography. The GAPDH level of cell lysates was assayed via Western blot. (**B**) For *MMP-9* mRNA expression, total RNA was prepared and analyzed using real-time PCR. (**C**) Cells were pretreated with Bay (0.1 μM) or 4p3f (1 μM) for 1 h and then incubated with LPS (10 μg/mL) for the indicated times. After treatment, cell lysates were collected and analyzed using Western blot for phosphorylation of p65 NF-κB (p-p65) and GAPDH (as an internal control). (**D**) The translocation of p65 NF-κB was determined by means of immunofluorescence staining in RBAs. Cells were pretreated with Bay or 4p3f for 1 h, and then stimulated with LPS for 30 min. Cells were fixed and labeled with an anti-p65 NF-κB antibody and a FITC-conjugated secondary antibody. Individual cells were imaged (scale bar = 50 μm) as described in Section 2. (**E**) Cells were transiently transfected with a promoter–reporter construct (pGL4.32-containing κB binding sites), pretreated with Bay (0.1 μM) or 4p3f (1 μM) for 1 h, and then stimulated with LPS (10 μg/mL) for the indicated times. After stimulation, the firefly luciferase activity of the promoter (κB) construct was measured as relative promoter activity to that of Renilla luciferase activity. Data are expressed as mean ± SEM (n = 3). * *p* < 0.05 and ** *p* < 0.01, as compared with the untreated control; ^#^ *p* < 0.05 and ^##^ *p* < 0.01, as compared with LPS-treated cells only. The images represent one of three individual experiments.

In the LPS signaling pathway, NF-κB binds to the promoter region of MMP-9 gene and modulates its expression [30]. To examine the role of NF-κB in LPS-induced MMP-9 expression in RBAs, cells were treated with or without the NF-κB inhibitor Bay11-7082 (Bay) for 1 h and then incubated with LPS (10 μg/mL) for 0, 16, or 24 h. The results showed that pretreatment with Bay (0.1 μM) significantly attenuated LPS-induced MMP-9 expression (Figure 3A). Additionally, pretreatment with 4p3f (1 μM) also suppressed LPS-induced *MMP-9* mRNA expression based on the real time-PCR analysis (Figure 3B). We further explored the effects of 4p3f on LPS-stimulated activation of NF-κB in RBAs, and the phosphorylation of p65 NF-κB (a subunit of NF-κB) was detected via Western blot. As shown in Figure 3C, LPS stimulated the phosphorylation of p65 NF-κB (p-p65) in a time-dependent manner, with a maximum increase at 30 min. We further found that LPS-stimulated p65 NF-κB phosphorylation was reduced by pretreatment with either Bay or 4p3f (Figure 3C). Hence, we further used immunofluorescence staining to assess the effect of 4p3f on LPS-stimulated NF-κB translocation from the cytoplasm into the nucleus in RBAs. The data showed that LPS could stimulate NF-κB translocation, and pretreatment with Bay or 4p3f both attenuated LPS-stimulated NF-κB translocation (Figure 3D). Next, to demonstrate whether LPS enhances NF-κB transcriptional activity, a promoter (containing κB binding sites)–luciferase reporter gene assay was used. The data showed that LPS increased NF-κB transcriptional activity in a time-dependent manner, with a maximum response after 4 h of incubation (Figure 3E). The transcriptional activity was attenuated by pretreatment with either Bay or 4p3f in RBAs. These results indicated that LPS-induced MMP-9 expression is mediated via the NF-κB-dependent pathway in brain astrocytes, and 4p3f can inhibit this response.

### 3.4. Effects of 4p3f on LPS-Induced STAT3-Mediated MMP-9 Expression in RBAs

**Figure 4:**
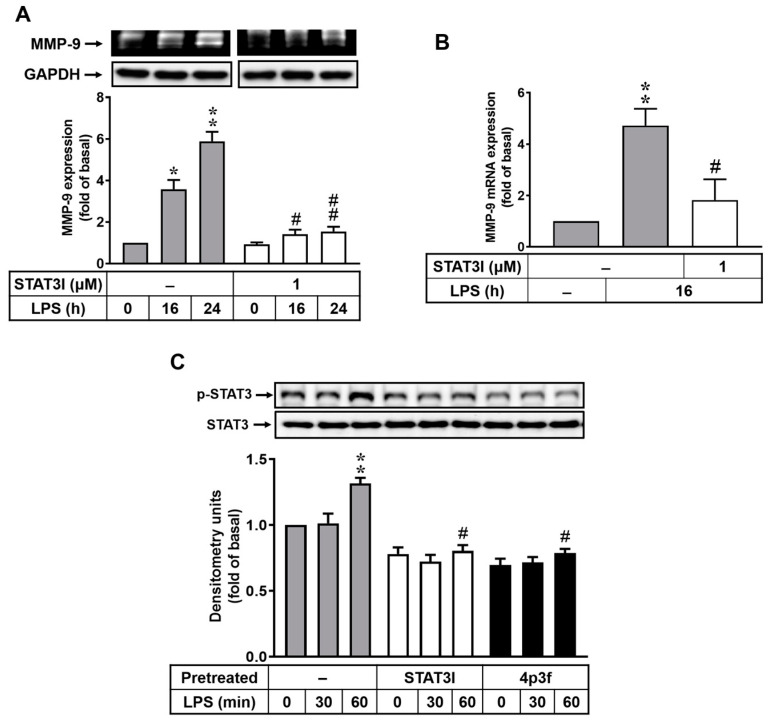
Effects of 4p3f on LPS-induced STAT3-mediated MMP-9 expression in RBAs. Cells were pretreated with or without STAT3I (1 μM) for 1 h and then incubated with LPS (10 μg/mL) for the indicated time intervals. (**A**) For MMP-9 expression, the conditioned media were collected and analyzed via gelatin zymography. The GAPDH level of cell lysates (as an internal control) was assayed by means of Western blot. (**B**) For *MMP-9* mRNA expression, total RNA was prepared and analyzed via real-time PCR. (**C**) Cells were pretreated with STAT3I (1 μM) or 4p3f (1 μM) for 1 h and then incubated with LPS (10 μg/mL) for the indicated times. After treatment, cell lysates were collected and analyzed via Western blot for phosphorylation of STAT3 (p-STAT3) and total STAT3. Data are expressed as mean ± SEM (*n* = 3). * *p* < 0.05 and ** *p* < 0.01, as compared with the untreated control; ^#^ *p* < 0.05 and ^##^ *p* < 0.01, as compared with LPS-treated cells only. The images represent one of three individual experiments.

In addition to NF-κB, STAT3 is known to regulate the induction of MMP-9 expression following stimulation by many factors in different types of cells [15,31,32]. Moreover, LPS-induced MMP-9 expression occurs via the STAT3 pathway in macrophages [31]. To investigate whether LPS-induced MMP-9 expression is mediated via a STAT3-dependent pathway, a STAT3 inhibitor (STAT3I) was used. As shown in Figure 4, cells were pretreated with STAT3I (1 μM) and then incubated with LPS (10 μg/mL) for the indicated time intervals. The data showed that pretreatment with STAT3I markedly attenuated LPS-induced upregulation of MMP-9 protein (Figure 4A) and *MMP-9* mRNA (Figure 4B). Furthermore, we observed whether LPS could stimulate STAT3 activation in RBAs, and the phosphorylation of STAT3 was detected using Western blot. The results showed that LPS could stimulate STAT3 phosphorylation, with a significant increase at 60 min (Figure 4C). Pretreatment with either STAT3I or 4p3f significantly inhibited LPS-stimulated phosphorylation of STAT3 in RBAs (Figure 4C). These results suggested that 4p3f attenuated LPS-induced MMP-9 expression by decreasing the activation of STAT3 in brain astrocytes.

### 3.5. MAPK-STAT3 Signaling Axis Is Crucial for LPS-Stimulated NF-κB Transcriptional Activity in RBAs

**Figure 5:**
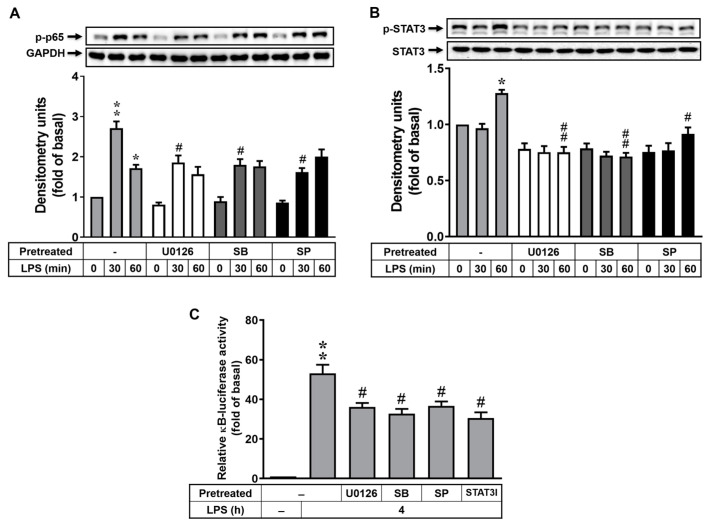
MAPK-STAT3 signaling axis is crucial for LPS-stimulated NF-κB transcriptional activity in RBAs. Cells were pretreated with U0126 (0.1 μM), SB202190 (1 μM), or SP600125 (0.1 μM) for 1 h and then incubated with LPS (10 μg/mL) for the indicated times. After treatment, cell lysates were collected and analyzed via Western blot for phosphorylation of p65 NF-κB (p-p65) (**A**) and STAT3 (p-STAT3 and total STAT3) (**B**), as well as GAPDH (an internal control). (**C**) Cells were transiently transfected with a κB-Luc reporter construct (pGL4.32). After transfection, cells were pretreated with U0126 (0.1 μM), SB202190 (1 μM), SP600125 (0.1 μM), or STAT3I (1 μM) for 1 h and then incubated with LPS (10 μg/mL) for 4 h. Cell lysates were collected and detected using a Dual-Luciferase^®^ Reporter Assay System. Data are expressed as mean ± SEM (*n* = 3). * *p* < 0.05 and ** *p* < 0.01, as compared with the untreated control; ^#^ *p* < 0.05 and ^##^ *p* < 0.01, as compared with LPS-treated cells only. The images represent one of three individual experiments.

In human monocytes, LPS triggers the activation of NF-κB through ERK1/2, JNK1/2, and p38 MAPKs [33]. In dendritic cells, LPS activates the STAT3 signaling pathway, which results in the recruitment of NF-κB and the expression of proinflammatory cytokine genes [34]. Thus, we explored the relationships between these signals. For the activation of MAPKs and NF-κB, cells were pretreated with U0126 (0.1 μM), SB202190 (1 μM), or SP600125 (0.1 μM) for 1 h and then incubated with LPS (10 μg/mL) for 0, 30, or 60 min. The phosphorylation of p65 NF-κB was detected using Western blot. As shown in Figure 5A, pretreatment with U0126, SB202190, or SP600125 significantly attenuated LPS-stimulated p65 NF-κB phosphorylation (p-p65), suggesting that LPS-stimulated activation of NF-κB is mediated in an MAPK (i.e., ERK1/2, p38, and JNK1/2)-dependent manner in RBAs. We further studied the relationships between MAPKs and STAT3. The results showed that pretreatment with U0126, SB202190, and SP600125 significantly decreased LPS-stimulated STAT3 phosphorylation (Figure 5B), suggesting that MAPKs (i.e., ERK1/2, p38, and JNK1/2) are also involved in LPS-stimulated STAT3 phosphorylation in RBAs. Moreover, we investigated the effects of the MAPK-STAT3 signaling axis on LPS-stimulated transcriptional activity of NF-κB in RBAs. As shown in Figure 5C, pretreatment with U0126, SB202190, SP600125, or STAT3I all markedly reduced κB-dependent promoter–reporter activity. Accordingly, these results demonstrated that LPS stimulated NF-κB transcriptional activity via an MAPK-mediated STAT3 signaling pathway in RBAs.

### 3.6. 4p3f Inhibits LPS-Induced MMP-9-Mediated Astrocytic Migration

**Figure 6:**
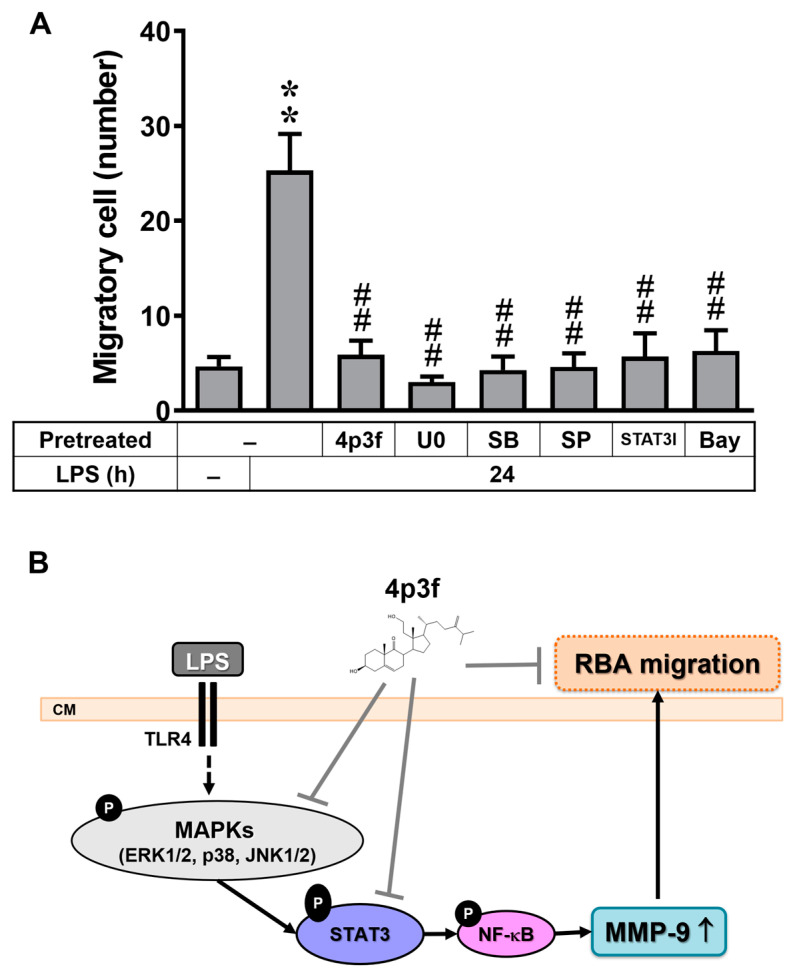
4p3f inhibits LPS-induced MMP-9-mediated astrocytic migration. (**A**) Cells were plated on 6-well culture plates, grew to confluence, and starved with a serum-free medium for 24 h. Cells were pretreated with U0126 (0.1 μM), SB202190 (1 μM), SP600125 (0.1 μM), STAT3I (1 μM), Bay (0.1 μM), or 4p3f (1 μM) for 1 h, and monolayer cells were manually scratched using a blue tip, as described in Section 2, and then incubated with LPS (10 μg/mL) for 24 h. Phase-contrast images of cells were taken at 24 h, and the number of cells that migrated was counted, as described in Section 2. Data are expressed as mean ± SEM (n = 3). ** *p* < 0.01, as compared with the untreated control; ^##^ *p* < 0.01, as compared with LPS-treated cells only. (**B**) Schematic presentation of the effects of 4p3f on LPS-induced MMP-9 expression and astrocytic migration. In brain astrocytes (RBAs), LPS induces STAT3 activation through MAPK (i.e., ERK1/2, p38, and JNK1/2) signals, resulting in NF-κB-dependent MMP-9 expression. The increased MMP-9 expression leads to RBA migration. 4p3f inhibits these LPS-induced MMP-9-mediated events (brain astrocytic migration) by suppressing the activation of MAPKs, STAT3, and NF-κB signaling pathways.

In brain astrocytes, we previously demonstrated that upregulation of MMP-9 participates in cell migration [35]. A recent study indicated that LPS induces MMP-9 expression and enhances cell migration, thereby contributing to the development of neuroinflammation [36]. Here, we further evaluated the effect of 4p3f on LPS-induced MMP-9-dependent RBA migration and its underlying mechanisms. Images of RBA migration were taken at 24 h after treatment with LPS (10 μg/mL) and observed. As shown in Figure 6A, LPS significantly triggered RBA migration, which was blocked by pretreatment with U0126 (0.1 μM), SB202190 (1 μM), SP600125 (0.1 μM), STAT3I (1 μM), or Bay (0.1 μM), indicating that LPS induces RBA migration via the MAPK (i.e., ERK1/2, p38, and JNK1/2), STAT3, and NF-κB cascade (Figure 6B). Furthermore, pretreatment with 4p3f also markedly inhibited LPS-induced RBA migration, demonstrating that 4p3f has an inhibitory effect on LPS-induced MMP-9-mediated RBA migration. These results confirmed that 4p3f have a protective effect on LPS-induced MMP-9-mediated brain inflammation by blocking the MAPK-STAT3-dependent NF-κB pathway in brain astrocytes.

## 4. Discussion

In the CNS, MMPs play a critical role in neurogenesis, neuronal network remodeling, and blood–brain barrier integrity [37,38]. And MMPs, especially MMP-9, are involved in the pathological processes of neuroinflammation, epilepsy, schizophrenia, autism spectrum disorder, multiple sclerosis, cerebral aneurysm, stroke, subarachnoid hemorrhage, Alzheimer’s disease, and Parkinson’s disease. The inhibition of MMP-9 has been proposed as a possible therapeutic strategy for the treatment of many CNS diseases [11,23,37,38,39,40]. Neuroinflammatory processes are critically involved in neurodegenerative conditions [41,42], with significant implications for the role of astrocytes. As the key cell type in the CNS, astrocytes actively participate in inflammatory and neuro-immunomodulatory responses [6,43]. The use of LPS as a potent proinflammatory agent is a well-established model of neuroinflammation in both in vitro and in vivo studies [12,36,44]. Previous studies have shown that LPS induces MMP-9 expression through various signaling mechanisms, including AP-1, FoxO1, and Nox/ROS-dependent NF-κB expression in brain astrocytes [13,14,36]. The RBA cell line, derived from dissociated cultures of normal neonatal rat brain tissues, is a widely recognized model of rat astroglial cells; it exhibits properties that closely resemble those of normal astrocytes, making it a valuable tool in research studies [12,36,44,45]. There are several steroids obtained from the genus *Sinularia* with anti-inflammatory ability and cytotoxicity on cancer cells [46,47,48,49]. In this study, we first report that a 9,11-secosteroid (4p3f), isolated from the Taiwan soft coral *Sinularia leptoclados,* exhibits inhibitory effects on LPS-induced MMP-9-mediated neuroinflammatory responses in rat brain astrocytes (RBAs). Notably, these responses include the regulation of MMP-9 expression and cell migration, and the underlying mechanism responsible for these effects involves the modulation of MAPK and STAT3 signaling pathways, as well as NF-κB activation. These results shed light on the potential therapeutic implications of 4p3f in several neuroinflammation-related brain disorders.

MMP-9 plays a significant role in CNS inflammation, and the development of an MMP-9 inhibitor holds promise as a therapeutic agent for both acute and chronic neuroinflammation [50]. The data from this study demonstrate that *MMP-9* gene expression in RBAs is induced by LPS in a time-dependent manner (Figure 1A,B). In Figure 1C,D, the marine sterol, 4p3f, exhibits anti-inflammatory activity in brain astrocytes by effectively inhibiting LPS-induced MMP-9 expression.

MAPKs, a family of serine/threonine protein kinases, play a pivotal role in inducing cellular responses to cytokines and external stress signals and regulating the production of inflammation mediators [27,51]. An increased MAPK activity in activated astrocytes leads to inflammatory and immune responses within the brain [52,53]. The MAPK family consists of serially activated kinases and the effector kinases ERK, JNK, and p38 MAPK [27,54]. Previous studies have shown that LPS stimulates MAPK activation [55,56,57], and activation of ERK1/2, JNK1/2, and p38 MAPK regulates MMP-9 expression and enzymatic activity in various types of cells [28,29,36,58]. Therefore, we investigated the role of MAPKs in LPS-induced MMP-9 expression in RBA cells. Our results demonstrate that ERK1/2, p38 MAPK, and JNK1/2 play a role in LPS-induced MMP-9 expression in brain astrocytes (Figure 2A,B). In addition, pretreatment of RBAs with 4p3f could attenuate LPS-induced phosphorylation of ERK1/2, p38 MAPK, and JNK1/2 (Figure 2C). However, the detailed mechanism underlying the attenuation of MAPK phosphorylation by 4p3f is challenging to delineate. We further used molecular docking analysis, and the data show that 4p3f binds to ERK1/2 directly (Figure S1A). All of these results are compatible with previous studies indicating that MMP-9 induction is mediated through the activation of MAPKs [29,36,58], and 4p3f may attenuate LPS-induced MMP-9 expression by interfering with MAPK signaling. However, the mechanism underlying the decreased LPS-induced phosphorylation of p38 MAPK and JNK1/2 by 4p3f pretreatment still needs to be elucidated.

Several studies have indicated that MMP-9 is induced in an NF-κB-dependent manner [59,60], and the NF-κB binding sites of the MMP-9 gene contribute to its expression upon LPS stimulation [30]. The data reveal that pretreatment with an NF-κB inhibitor significantly suppresses LPS-induced MMP-9 expression in RBA cells (Figure 3A,B). Additionally, both pretreatment with 4p3f and pretreatment with the NF-κB inhibitor effectively eliminate LPS-stimulated NF-κB phosphorylation (Figure 3C, p-p65) and translocation (Figure 3D), and reduce its transcriptional activity in the κB-promoter–reporter assay (Figure 3E). These findings suggest that the inhibitory effect of 4p3f on LPS-induced MMP-9 expression is likely attributed to the suppression of NF-κB activation in RBA cells.

STAT3 plays a critical role in the pathogenesis of neuroinflammation and Alzheimer’s disease [61]. Moreover, it has been reported that the activation of STAT3 regulates MMP-9 production [31,32]. Here, we further observed the role of STAT3 in LPS-induced MMP-9 expression in RBA cells. As shown in Figure 4, pretreatment with a STAT3 inhibitor (STAT3I) significantly attenuated the LPS-induced MMP-9 increase and STAT3 phosphorylation in RBA cells. Additionally, pretreatment with 4p3f also inhibited LPS-stimulated phosphorylation of STAT3 in RBA cells (Figure 4C). Furthermore, 4p3f could directly bind to STAT3, as shown in the molecular docking analysis (Figure S1B). These results suggest that the STAT3 signaling pathway may play a critical role in the regulation of *MMP-9* gene expression during inflammatory responses, whereby 4p3f attenuates MMP-9 expression by inhibiting the STAT3 pathway.

TLR4 acts as a receptor for LPS, and TLR signaling activates the downstream of MAPKs, NF-κB [62], and STAT3 [63]. Moreover, LPS stimulation triggers the activation of NF-κB through the MAPK signaling pathway [33] and the STAT3 signaling pathway [34]. Herein, we further investigated the crosstalk between the MAPK, NF-κB, and STAT3 signaling pathways in LPS-activated RBA cells. The data indicate that MAPKs (ERK1/2, p38, and JNK1/2) are involved in LPS-stimulated NF-κB and STAT3 activation (Figure 5A,B). Furthermore, our findings suggest that LPS-enhanced NF-κB transcriptional activity may occur through MAPK (ERK1/2, p38, and JNK1/2) and STAT3 signals (Figure 5C). These results provide evidence for the complex interplay between these signaling pathways and their roles in regulating the inflammatory responses in RBA cells upon LPS stimulation.

Finally, the upregulation of MMP-9 expression in brain astrocytes and its consequent enhancement of cell migration contribute to the development of neuroinflammation and neurodegenerative diseases [33,64,65], such as AD. AD is a progressive neurodegenerative disorder with synaptic loss as an early event in disease development. Synaptic loss and dysfunction contribute to the cognitive deficits seen in AD patients. Cerebrovascular disruption of the BBB leads to neuronal damage and inflammation. At an early time point of BBB dysfunctions, MMP-9 is actively engaged in response to a neuronal injury with astrocytic activation. Although MMP-9-induced astrocyte migration plays a role in forming a glial scar and lesion repair [8], the activated MMP-9 may also disrupt the neurovascular unit by altering synaptic plasticity and leading to cognitive decline. As shown in Figure 6A, we observed that LPS-triggered RBA cell migration was effectively blocked by pretreatment with 4p3f, as well as by pretreatment with inhibitors of MAPKs (ERK1/2, p38, and JNK1/2), STAT3, or NF-κB. These results collectively indicate that 4p3f exhibits both anti-inflammatory and antimetastatic activities in LPS-induced MMP-9-mediated events in RBA cells. The findings suggest that the marine sterol 4p3f may be a potential therapeutic agent for the treatment of neuroinflammatory and neurodegenerative disorders.

In conclusion, we investigated the in vitro effects of 4p3f, a sterol isolated from the soft coral *Sinularia leptoclados*, on anti-inflammatory and anti-metastatic activities in brain astrocytes. 4p3f protects against LPS-induced damage by attenuating the signaling pathway involved in MMP-9 expression and cell migration in rat brain astrocytes (RBA cells). We further used various signaling inhibitors to demonstrate that 4p3f protects against LPS-induced MMP-9-mediated RBA cell migration by inhibiting the MAPK (ERK1/2, p38, and JNK1/2), STAT3, and NF-κB cascade (Figure 6B). Pharmacological approaches suggest that research on 4p3f may yield useful therapeutic targets for the treatment of brain inflammatory diseases. In the future, in vivo experiments, such as animal or organoid models, should be tested to explore the protective effects of 4p3f on neuroinflammatory events.

## Data Availability

The data obtained from this study are available in this article.

## Supplementary Materials

The following supporting information can be downloaded at: https://www.mdpi.com/article/10.3390/biomedicines12010226/s1, Figure S1: Molecular docking about 4p3f and the proteins of ERK1/2 (A) or STAT3 (B). Two-dimensional binding modes and three-dimensional H-binding modes showing the interactions between 4p3f and ERK1/2 or STAT3 protein.
