# Supplementary material for: A Brain-Protective Sterol from Soft Coral Inhibits Lipopolysaccharide-Induced Matrix Metalloproteinase-9-Mediated Astrocytic Migration
Source: Biomedicines. 2024 Jan 19;12(1):226. doi: 10.3390/biomedicines12010226 (PMC10813456; doi:10.3390/biomedicines12010226)
Supplement: Supplementary file 1 [file biomedicines-12-00226-s001.zip › biomedicines-2764893-Supplementary data(R3) 1.pdf]

Supplementary information

Method

Molecular Docking

The molecular docking software used here was Discovery Studio 2021 Client. The preparation process of the target protein as follows: The crystal structure of the target proteins of ERK1/2 (PDB ID: 6G9M) and STAT3 (PDB ID: 6NJS) were downloaded from the PROTEIN DATA BANK (https://www.rcsb.org/). The grid boxes after identifying the binding site of the protein and its residues were created. We visualized and analyzed the ligand-protein complexes and used the LibDock scores to predict binding affinities between 4p3f and target proteins, ERK1/2 and STAT3.

Result

Here, we further explored the interaction modes between 4p3f and ERK1/2 or STAT3 protein by DS molecular docking. The molecular docking data indicated that 4p3f can interact with the key protein of ERK (Figure S1A), suggesting that 4p3f could inhibit LPS-induced MMP-9-mediated event (cell migration) in RBA via blocking ERK-dependent pathway. Moreover, the molecular docking data also indicated that 4p3f can interact with the key protein of STAT3 (Figure S1B), suggesting that 4p3f can affect STAT3-mediated signaling pathway.

A.

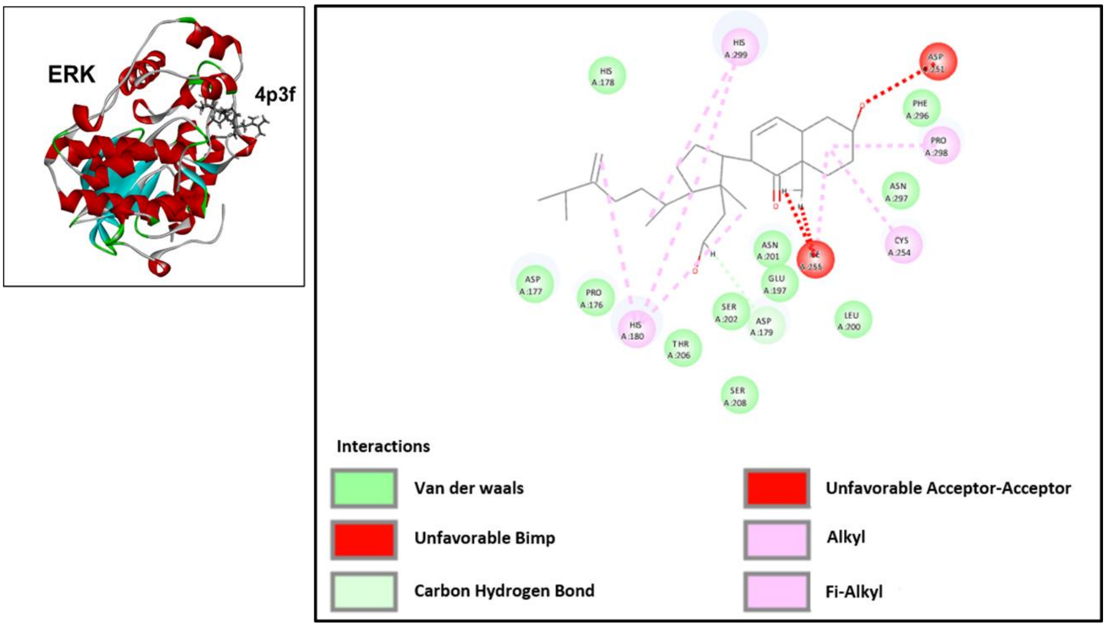

B.

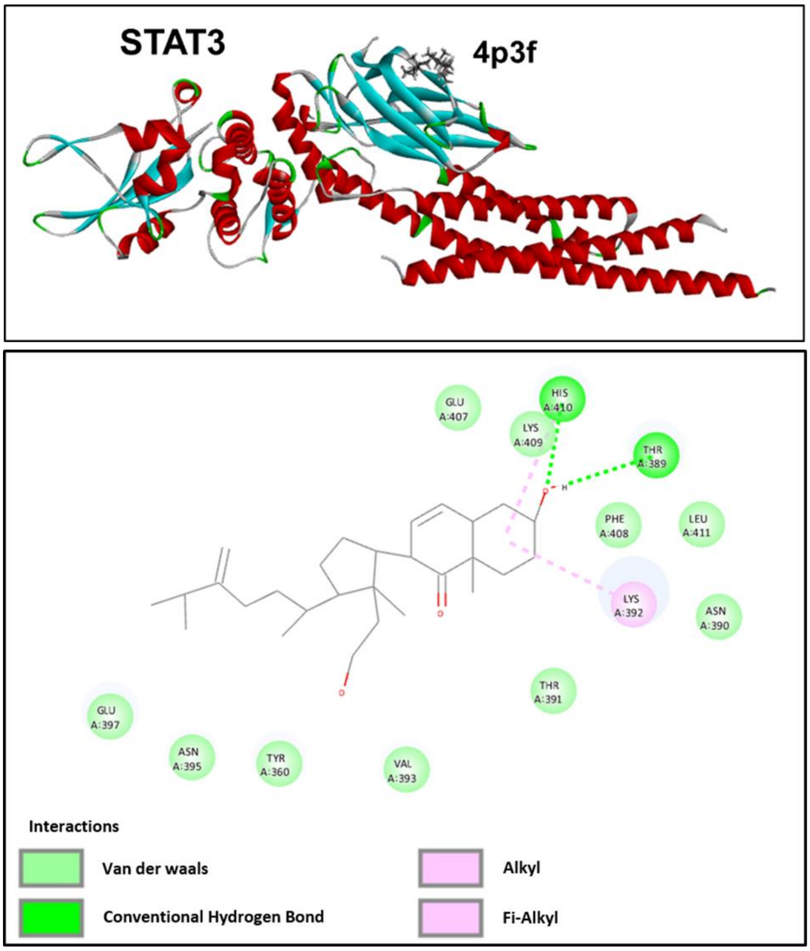

Figure S1. Molecular docking about 4p3f and the proteins of ERK1/2 (A) or STAT3 (B). Two-dimensional binding modes and three-dimensional H-binding modes showing the interactions between 4p3f and ERK1/2 or STAT3 protein.
